# Distinctive ocean interior changes during the recent warming slowdown

**DOI:** 10.1038/srep14346

**Published:** 2015-09-23

**Authors:** Lijing Cheng, Fei Zheng, Jiang Zhu

**Affiliations:** 1 International Center for Climate and Environment Sciences, Institute of Atmospheric Physics, Chinese Academy of Sciences, 100029, Beijing, China

## Abstract

The earth system experiences continuous heat input, but a “climate hiatus” of upper ocean waters has been observed in this century. This leads to a question: where is the extra heat going? Using four *in situ* observation datasets, we explore the ocean subsurface temperature changes from 2004 to 2013. The observations all show that the ocean has continued to gain heat in this century, which is indicative of anthropogenic global warming. However, a distinctive pattern of change in the interior ocean is observed. The sea surface (1–100 m) temperature has decreased in this century, accompanied by warming in the 101–300 m layer. This pattern is due to the changes in the frequency of El Niño and La Niña events (ENSO characteristics), according to both observations and CMIP5 model simulations. In addition, we show for the first time that the ocean subsurface within 301–700 m experienced a net cooling, indicative of another instance of variability in the natural ocean. Furthermore, the ocean layer of 701–1500 m has experienced significant warming.

Global surface temperature observations indicate a slowdown of the earth surface warming within this century^1^,^2^. This slowdown, commonly referred to as a “hiatus period”^3^,^4^, has led to a spirited debate in the scientific community about whether global warming has slowed or even stopped^5^. Several explanations have been proposed for the recent observations. Model-based studies suggest that the natural variability is large enough to account for the entirety of the recent climate hiatus because the Inter-decadal Pacific Oscillation (IPO) has exhibited an altered phase since the late 1990s^6^,^7^ and has cooled the sea surface in the tropical Pacific Ocean, which controls the global surface temperature^7^,^8^. Other mechanisms have also been examined and are thought to contribute a smaller portion of the recent climate change slowdown. For instance, insufficient data coverage in the arctic is responsible for part of the global surface temperature slowdown^9^, suggesting that a rigorous examination of horizontal sampling of *in situ* observations is needed^10^. Volcanic forcing is thought to serve as a negative forcing on the climate system^11^ and to result in a cooling of the earth’s surface. Furthermore, changes in the solar cycle^12^, aerosols^12^,^13^ and the stratosphere^14^ have been examined and are considered to have contributed to the recent climate change slowdown, but they have been considered to have made a minor contribution.

However, the authors of ref. 15 hold another point of view. They suggested that the change in salinity-driven Atlantic Ocean circulation was responsible for the recent climate hiatus. By using state-of-the-art ocean models, ref. 16 linked the heat decrease during the recent slowdown in the equatorial Pacific Ocean, North Atlantic Ocean and Southern Ocean to unique mechanisms in each region.

Although numerous explanations have been proposed, one key question related to the climate hiatus remains: taking into account that energy is continuously put into the earth system due to the continuous release of carbon dioxide and the consequent increase in the atmospheric greenhouse effect^17^, where has the heat gone? Observations^18^ and re-analysis data^19^ show that the deep ocean (700–2000 m) has warmed in the current century, suggesting that the heat may be transferred into the deeper ocean. An examination of climate models also indicates that excess heat has been transferred into the subsurface ocean (deeper than 150 m^6^ or 300 m^4^) as a result of the anomalous trade winds in the tropical Pacific Ocean since the 1990s.

Therefore, a detailed analysis on ocean interior changes based on *in situ* temperature observations is crucial to understand the recent slowdown. Here, we present a complete map of the ocean subsurface temperature change based on *in situ* ocean subsurface observations that depict the ocean subsurface heat inventory. Such mapping is possible because the ocean observation network has achieved global coverage in this century (since 2004) through the Argo project^20^,^21^ together with traditional ship-based observations, increasing the confidence in estimates of the change in ocean temperature. In addition, attaining an understanding of the mechanisms of the observed signals is essential. Climate models could provide an effective and powerful way to accomplish this task. Consequently, such models are also used in this study to understand the recent slowdown.

## Results

### Ocean temperature change at upper 300 m

**Figure 1:**
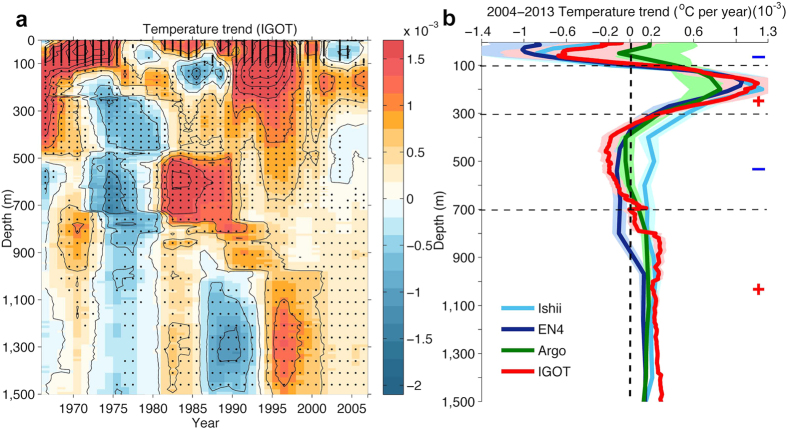
Decadal ocean temperature trend. (**a**) The global-averaged ocean temperature trend at each depth from 1 to 1500 m is calculated for every 10 years starting from 1966 and using *in situ* observations (IGOT). The trend shown at year N in x-axis is from year N to N+9 (N to N+8 at year 2005, N to N+7 at year 2006). Values significant at the 95% confidence interval are denoted by black dots. (**b**) The global-averaged ocean temperature trend from 2004 to 2013 at depths from 1 to 1500 m and calculated by using four observation products. 90% confidence intervals are presented in shades.

Figure 1 presents the 10-year temperature trends at each depth, from the sea surface to 1500 m, starting from 1966 (based on the IGOT dataset discussed in the Methods section), with 95% significant signals highlighted in black dots. Due to the lack of *in situ* subsurface observations prior to 2000, which could bias the trend calculation, in this study, we focus on the ocean changes in this century. Since 2000, nearly coincident with the onset of the climate hiatus, the ocean surface has been cooling (we define such a period as an “SST-Hiatus period”). This cooling persists down to 100 m in the ocean and is accompanied by a significant ocean warming at 101–300 m (Fig. 1a,b). The same structure can also be found in the three observational products^22^,^23^, as shown in Fig. 1b and Figure S1. Argo data show near-zero trends in the upper 100 m, similar to ref. 24, although the 90% confidence intervals overlap with the other datasets. It is worth noting here that due to the lack of Argo data on the Western Pacific Ocean, particularly the Indonesian Archipelago, the ocean heat content trend based on Argo data would be biased, according to a recent study^25^. This bias may be partly responsible for this contradiction. Choices related to quality-control strategies, mapping strategies^26^, climatologies^27^, and XBT bias correction schemes^26^ by the data producers could also contribute to the differences in these observed estimates.

It is also apparent that the global ocean change is controlled by the Pacific Ocean (Figure S2), while the Atlantic Ocean shows cooling in the upper 400 m (Figure S2). This is in contrast to a recent paper by ref. 15, which implied Atlantic control of the recent slowdown. The contradiction is possibly due to the difference in the choice of analysis period: 1999–2012 by ref. 15 instead of 2004–2013 by this study. Large uncertainty in the OHC calculation is found in the late 1990s and early 2000s when comparing different OHC estimates by different groups^28^,^29^,^30^. This results in inconsistent OHC trends among different datasets. A recent study^10^ implied that the observation transfer from a ship-based system to an Argo-based system in the early 2000s could result in artificial OHC signals, the magnitude of which is sensitive to the mapping methods. For this reason, we analyze the period 2004–2013 in this study to exclude the impact of changes in the observation system. The Indian Ocean experiences consistently rapid warming from 1 m to 1500 m (Figure S2). Ref. 31 reported an abruptly increasing OHC in the Indian Ocean during the recent climate slowdown that originates from Pacific Ocean via Indonesian throughflow. Ref. 32 suggests that the rapid warming in the Indian Ocean is controlled by the Pacific Ocean due to El Niño–Southern Oscillation (ENSO) teleconnections. Ref. 33 also indicates that the Atlantic Ocean change is remotely controlled by the tropical Pacific via quasi-stationary atmospheric Rossby waves. However, ref. 16 suggests that there are different mechanisms responsible for the changes at different ocean basins. These contradictions imply that the relation among the three major basins during the recent climate hiatus has yet to be understood.

**Figure 2:**
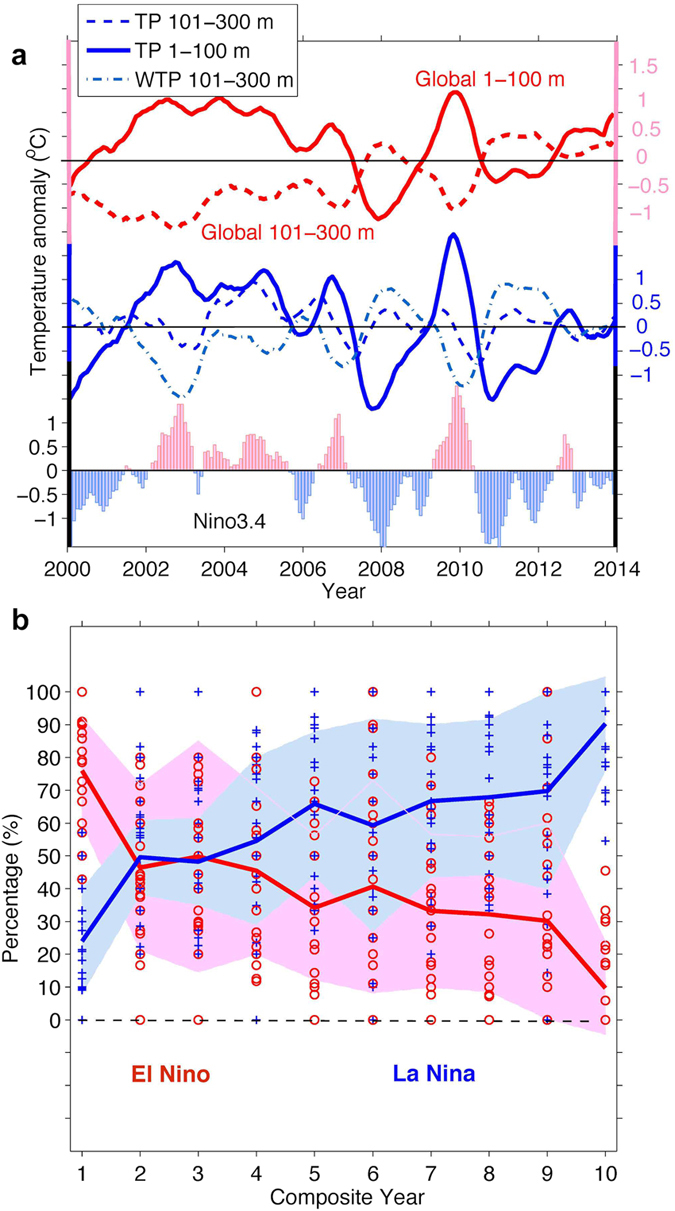
Global and regional ocean temperature change and ENSO variability. (**a**) The temperature anomaly at 0–100 m (solid) and 101–300 m (dashed) for the Global Ocean (red), Tropical Pacific (TP, 5 °S–5 °N) (blue), and tropical Western Pacific (TWP, chain line). All the time series are normalized by their standard deviation. The Niño3.4 index is shaded where the Oceanic Niño Index (ONI) is used (http://www.cpc.ncep.noaa.gov/products/analysis_monitoring/ensostuff/ensoyears.shtml). (**b**) Percentage of the occurrence of El Niño events (red) and La Niña events (blue) in SST-Hiatus periods estimated from the CMIP5 models; the ensemble mean of 23 model results is presented as a solid line with shading showing the spread of ±1 standard deviation.

It also remains to be understood why the ocean layers from 1–100 m are cooling and the layers from 101–300 meters are warming. It is possible that these changes are linked. Recent studies indicate that the global surface temperature change is controlled by the sea surface temperature in the tropical Eastern Pacific^8^,^34^ and that accurate simulations of the El Niño/Southern Oscillation (ENSO) cycle help in reconstructing the recent slowdown^35^. Figure 2a presents the monthly mean global ocean temperature change for 1–100 m and 101–300 m compared with the temperature anomalies in the tropical Pacific Ocean. It appears that the global-ocean temperature change from 1–100 m is significantly (95% confidence) correlated with the signals in the tropical Pacific (*r* = 0.93) and the Niño3.4 index (*r* = 0.86). This high correlation confirms the Pacific tropical control of the global upper ocean temperature change, which is dominated by ENSO variability. For 101–300 m, the global temperature change is mostly controlled by the tropical Western Pacific (*r* = 0.77), which is also dominated by ENSO variability; this condition is the opposite for the Niño3.4 index (*r* = −0.79). Correlations between the global temperature changes for both 1–100 m and 101–300 m with the local temperature change also show significant values at low latitudes with a similar pattern of ENSO variability, as shown in Figure S3.

Why does a different trend appear at 1–100 m and 101–300 m even though these ocean layers are both linked to ENSO? According to the NOAA oceanic Niño index (http://www.cpc.noaa.gov/products/analysis_monitoring/ensostuff/ensoyears.shtml), which is defined as a three-month running mean of SST anomalies in the Nino-3.4 region (5 °N–5 °S, 120–170 °W), an El Niño (La Niña) event is identified when five consecutive months of the ONI index are larger (smaller) than 0.5 °C (−0.5 °C). Based on this definition, there was a series of El Niño events at the beginning of this century—in 2002/03, 2004/2005, 2006/07 and 2009/10—but subsequent La Niña events have occurred recently, such as in 2005/06, 2007/08, 2008/09, 2010/11, and 2011/2012. A superposition of time-wise ENSO evolution and layered ocean heat content changes is shown in Fig. 2a. El Niño events are tied to warmer conditions at the upper ocean (i.e., 0–100 m) in the tropical Pacific but colder conditions below 100 m (i.e., 101–300 m)^34^. La Niña events induce the opposite changes as those induced by El Niño events. This effect is due to the dipole-like structure along the thermocline in the tropical Pacific during ENSO cycles^34^. Therefore, from 2002 to 2012, the upper ocean (1–100 m) in the tropical Pacific transferred from a warmer condition to a colder condition, resulting in decreasing temperatures, whereas the ocean at 101–300 m transferred from a colder condition to a warmer condition, leading to warmer conditions. Therefore, it is evident that changes in El Niño and La Niña event frequency have contributed to the recent slowdown.

In addition, the seasonal cycle of the slowdown period (the slowdown occurs in winter at 1–100 m and in summer at 101–300 m) supports this hypothesis (Figure S4) because ENSO develops into the mature phase (peak) in winter near the sea surface (i.e., 1–100 m), but subsurface signals occur several months earlier in the Western Pacific^36^. Such a change in the Pacific Ocean dominates the global changes, as suggested by our observational results and a recent study^33^.

**Figure 3:**
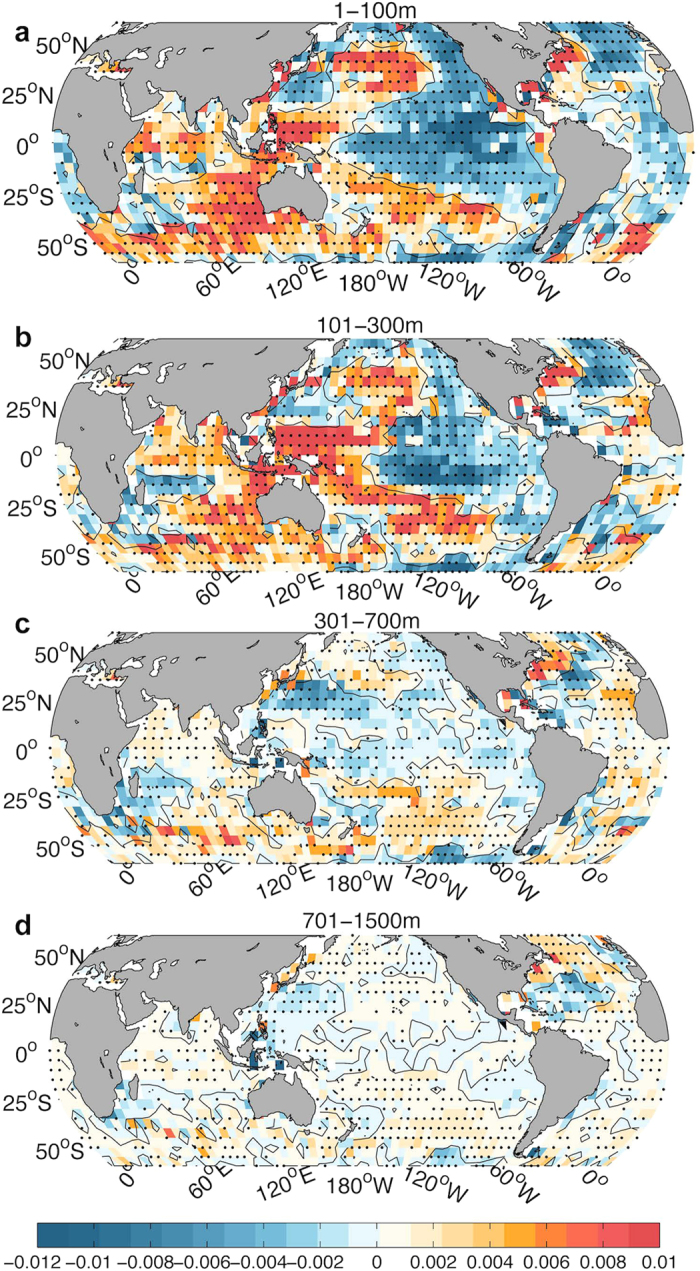
Geographical distribution of ocean temperature trend from 2004 to 2013 based on *in-situ* observations (IGOT) at different depths. (**a**) 1–100 m; (**b**) 101–300 m; (**c**) 301–700 m; (**d**) 701–1500 m. Trends significant at the 95% confidence interval are denoted by black dots. This map was generated using MATLAB.

To further reinforce this conclusion, we examined simulations of pre-industrial controls run from 23 global-coupled climate models of the Coupled Model Inter-comparison Project Phase 5 (CMIP5)^37^. In each model, we detected the SST-Hiatus periods, which are defined as 10-year periods with a negative SST trend. Then, we counted the number of El Niño and La Niña events within the SST-Hiatus periods. In Fig. 2b, all the models show that there are more occurrences of El Niño events at the beginning of the SST hiatus period; however, more La Niña events appear in the latter part of the period. On average, in the first year of the SST-Hiatus period, 80% (20%) of ENSO events are El Niño (La Niña) events, but the frequency of El Niño (La Niña) events decreases (increases) to 10% (90%) in the last year. This pattern leads to the conclusion that the SST-Hiatus is closely tied to changes in the frequency of El Niño and La Niña events (ENSO characteristics). Recharge-discharge^38^ provides a possible explanation for ocean changes in the Pacific Ocean during the SST-Hiatus period. Because heat is transferred to off-equator regions during La Niña events, it appears as cooling near the equator and warming in the subtropics, as indicated in Fig. 3.

Although both observations and models provide evidence showing a link between ENSO states and the recent slowdown, the origin of the change in ENSO is still unknown^39^. Studies have suggested that decadal variability (i.e., IPO) plays a dominate role in the recent climate hiatus^3^,^4^,^6^,^40^. Ref. 7 examine the drivers of hiatus decades in CMIP5 models (historical and projected) and find a distinct IPO (or low-pass filter ENSO) signal in hiatus and accelerated warming decades. It is possible that the IPO phase change induced the changes in ENSO statics. Moreover, studies^41^,^42^,^43^ suggest that a warming Indian Ocean can weaken El Niño during its developing and terminating phases. Ref. 43 illustrated the dominant contribution of the basin-wide Indian Ocean warming in the fast transition of ENSO during spring and early summer. Ref. 42 attributed this fast transition to anomalous wind flow over the west and central Pacific Ocean, forced by Indian Ocean warming, which ultimately modulates the propagation of the upwelling thermocline anomaly and regulates the ENSO events.

### Ocean temperature change at 301–700 m depths

How has the deeper (301–700 m) ocean changed during the recent SST-Hiatus period? Are these changes linked to the ocean heat content variability in the upper 300 m? The observations in Fig. 1b show that the ocean from 300–700 m follows a weak cooling trend from 2004 to 2013 (also shown in Figure S1), with the only exception being the Ishii data used in a recent study^15^. Moreover, the geographical distribution of the temperature trend from 2004 to 2013 at 301–700 m shows a distinguishable pattern compared with the upper 1–100 m and 101–300 m, as shown in Fig. 3. A band-like cooling is shown at 301–700 m within 20 °S–30 °N in the Pacific Ocean. In contrast, the ocean subsurface for both 1–100 m and 101–300 m experienced a La-Niña-like pattern. Therefore, we suspect that a different mechanism is responsible for the ocean variability at 301–700 m compared with that in the upper 300 m.

**Figure 4:**
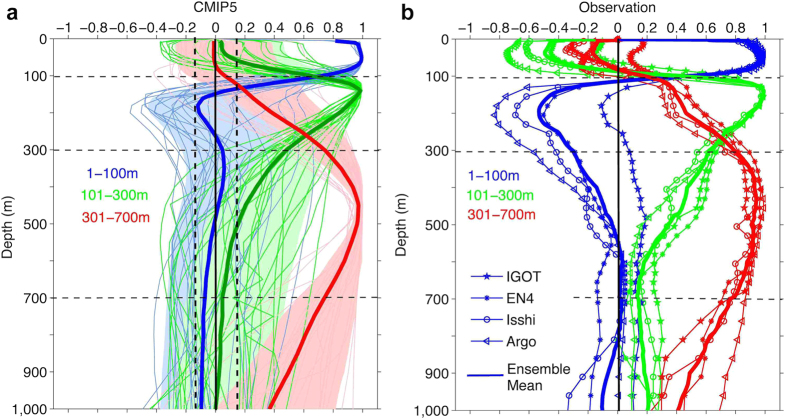
Correlations of ocean temperature changes among different depths. (**a**) Correlations of global-averaged temperature change among 1–100 m (blue), 101–300 m (green), 301–700 m (red) and each vertical model level using 23 CMIP5 simulations. The ensemble means of all models are shown as solid lines with a spread of ±1 standard deviation. (**b**) The same as (**a**) but using four observation products.

To further investigate this issue, we calculated the correlation between the global temperature change at 301–700 m with that at each model level based on both CMIP5 simulations and *in situ* observation products (Fig. 4a,b). All the models and observations indicate minimum correlations between the upper 300 m and 301–700 m temperature variation, implying independent ocean variability at the two depth intervals. An examination of the correlation between the global mean temperature at the 301–700 m depth and changes in the local temperature does not reveal an ENSO-like pattern, as shown in Figure S3, indicating that the changes in the 301–700 m depth may not be linked to ENSO variability. By contrast, when calculating correlations between global temperature changes for 1–100 m with those at each model level, the results show a maximum positive correlation at 0–100 m and a significant negative correlation at 101–300 m (Fig. 4). These results are mirrored when calculating the 101–300 m correlations with model depths. This finding again confirms that the 1–100 m and 101–300 m depths are linked but are always in different phases. However, temperature changes in the deeper (301–1500 m) ocean have a weak correlation with both the 1–100 m and 101–300 m temperature change, indicating that the SST-Hiatus might be limited in the upper 300 m. This finding also disagrees with ref. 15, where the authors suggested a much deeper appearance of the recent climate hiatus.

It is also possible that ENSO variability has some delayed impacts on 301–700 m ocean changes. We conducted a time-lag (from −36 to 36 months) correlation analysis based on EN4 and Ishii data between the temperature change at 0–20 m (representative of SST) and different depths (i.e., 21–100 m, 101–300 m, 301–700 m), as shown in Figure S5. The results indicate that there is a significant simultaneous positive correlation at the upper 100 m (r ∼ 0.8) and a negative correlation at 101–300 m (r ∼ −0.6) with SST, indicating the ENSO signals. However, there is a very weak simultaneous or lag correlation (−0.3 < r < 0.3) between SST and the 301–700 m averaged temperature change. The results indicate again that the SST-Hiatus may be limited in the upper 300 m. However, we should note that this test still cannot reject the possibility that ocean variability at the upper 300 m has an impact on the deeper ocean on a decadal scale or by nonlinear processes. Answering this question requires much longer temperature records than 50 years, which are not yet available.

Therefore, it is likely that there are two independent modes of natural variability that have caused the ocean to experience two hiatus periods in the present century: the SST and the 301–700 m layer. We named the period with ocean cooling from 301–700 m as the “301–700 m Slowdown” period (i.e., there is no significant trend within 301–700 m). We did not name it as another hiatus period because the subsurface temperature variations are determined by various complex ocean dynamics, such as thermocline ventilation, recharge-discharge, and meridional overturning circulation. However, the flattening of the SST trend has special significance because it can directly affect the coupled climate response. The subtropical thermocline ventilation in the Pacific might be a reason for the 301–700 m Slowdown, which could induce a lower-thermocline temperature change.

**Figure 5:**
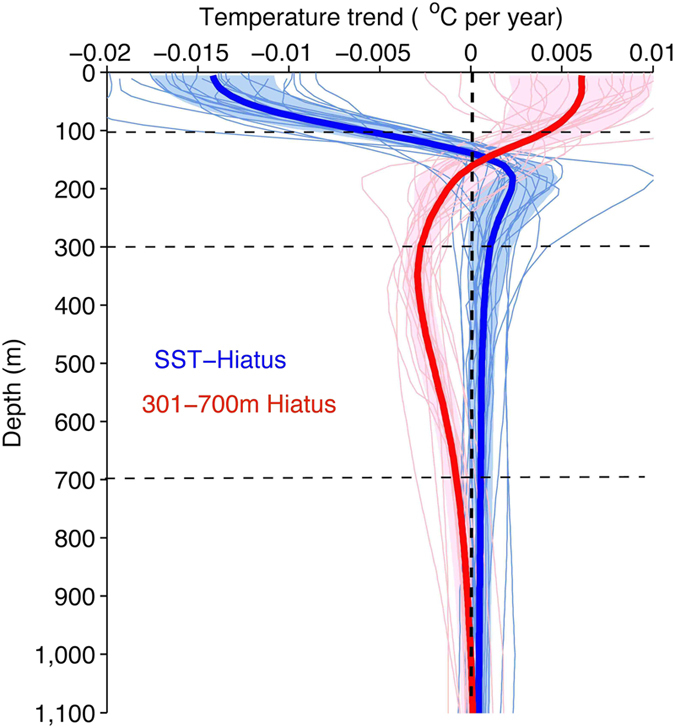
Temperature trend at depths in two types of hiatuses. Global-mean ocean temperature trends in SST-Hiatus periods (blue) and in 301–700 m Slowdown periods (red) based on models from CMIP5 simulations. The shades show the area of the ±1 standard deviation.

Identification of the 301–700 m Slowdown periods can also be made in the CMIP5 model simulations by constructing a composite for the 301–700 m Slowdown for each model. The 10-year trends of global ocean temperature at each depth are presented in Fig. 5, which shows the ocean cooling from 170–700 m but warming slightly from 1–170 m. This is distinctive from the subsurface variation during the SST-Hiatus period. During the SST-Hiatus period, the ocean experienced a significant cooling from 1–150 m and warming from 151–300 m, but there was no significant trend below 300 m for the global average. This global average is also dominated by the Pacific Ocean (Figure S6).

**Figure 6:**
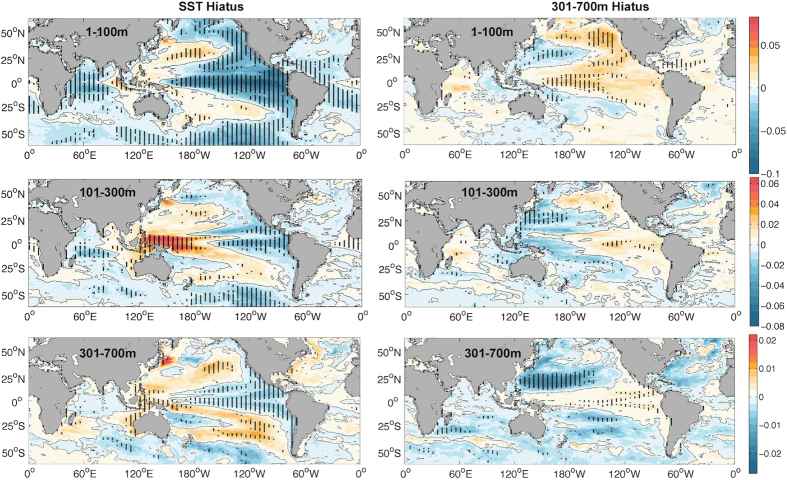
Ensemble mean of temperature trend for 23 CMIP5 models during the SST-Hiatus period (left side) and 301–700 m Slowdown period (right side). Top: 1–100 m averaged temperature trend, Middle: 101–300 m; Bottom: 301–700 m. The contour is 0 °C/yr in black. The units are °C/yr. Values significant at the 95% confidence interval are denoted by black dots. This map was generated using MATLAB.

**Figure 7:**
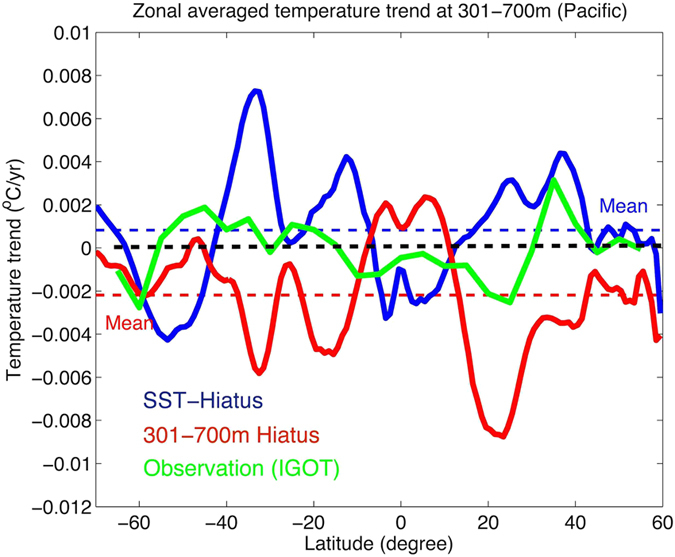
Zonal averaged temperature trend at 301–700 m in the Pacific Ocean. The ensemble means of the 23 models from CMIP5 are presented here: SST-Hiatus in blue and 301–700 m Slowdown in red. The same result from 2004 to 2013 calculated by using IGOT data is shown in green.

Local temperature trends in the model composite of both the 301–700 m Slowdown and the SST-Hiatus period are presented in Fig. 6. The model composite for the SST-Hiatus period is broadly consistent with observations (compare with Fig. 3) at 1–300 m in the Pacific, indicating the appearance of the SST-Hiatus in this century in the upper 300 m. The only exception occurs within ∼20–30 °N in the Western Pacific, which shows cooling trends in the observations. However, this feature is consistent with the 301–700 m Slowdown composite (Figs 6 and 7), implying that the SST-Hiatus and the 301–700 m Slowdown occurred simultaneously in the recent decade. In addition, the 301–700 m Slowdown composite shows a broad cooling trend in the Northern Pacific at 301–700 m, with a peak at 20–30 °N (Figs 6 and 7), which is consistent with observations (Fig. 3, Figure S7). It is likely that the middle latitudes of the Northern Pacific are a crucial area for the 301–700 m Hiatus and are the predominant site of the 301–700 m change in this century.

In the Pacific Ocean, we compare the observational trend of temperature for 301–700 m with the model composite at the same depth interval in Fig. 7. The results show that the observational changes are most likely contributed by the two variabilities: SST-Hiatus and 301–700 m Slowdown. The observed ocean changes within 20 ^o^N–30 ^o^N are more influenced by changes represented by the 301–700 m Slowdown, while the other regions are more indicative of ocean changes represented by the SST-Hiatus.

## Discussion

**Figure 8:**
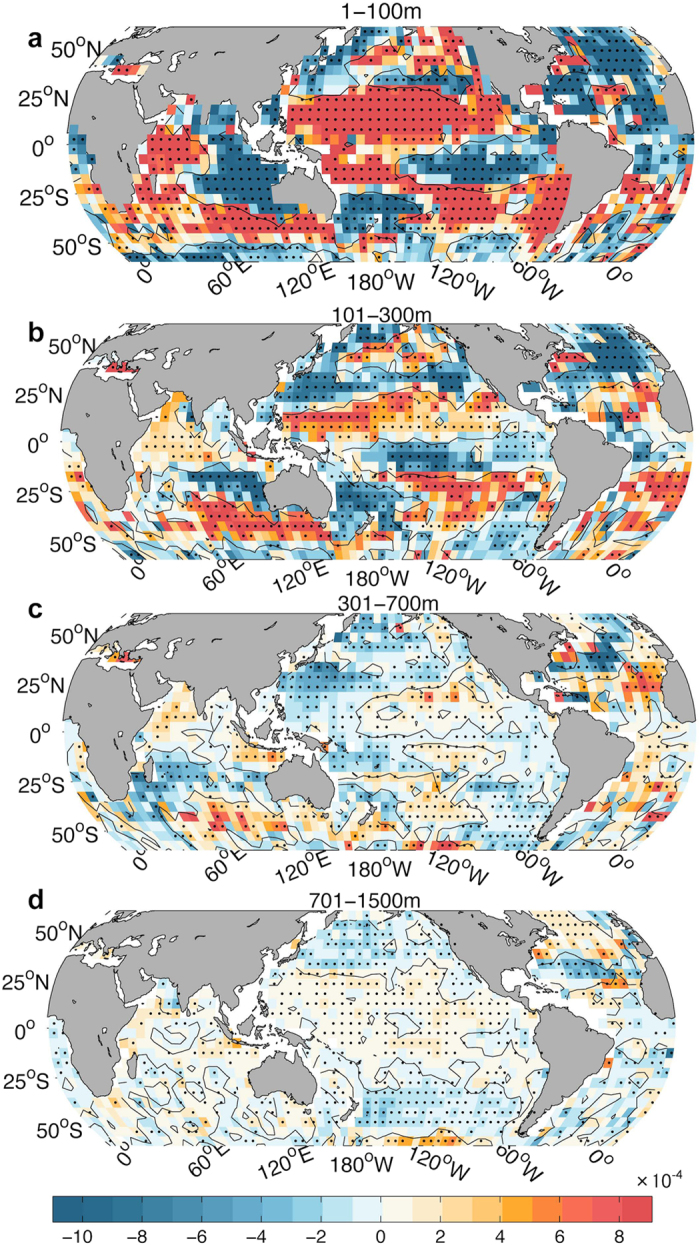
Geographical distribution of ocean salinity trend from 2004 to 2013 based on WOD13 at different depths. (**a**) 1–100 m; (**b**) 101–300 m; (**c**) 301–700 m; (**d**) 701–1500 m. Trends significant at the 95% confidence interval are denoted by black dots. This map was generated using MATLAB.

*In situ* observations provide us with an insightful depiction of what occurred in the ocean interior during the recent slowdown. Two slowdowns, the SST Hiatus and the 301–700 m Slowdown, are observed. They are likely linked to two independent natural modes of variability, but they have occurred coincidently since 2000. The SST-Hiatus is significantly linked to changes in the ENSO characteristics, which are responsible for the heat redistribution between the 1–100 m and 101–300 m layers. The salinity change pattern during this time period also reveals ENSO fingerprints (Fig. 8). The ocean variability for water depths of 301–700 m during the Slowdown period, which has never been reported by previous studies due to a lack of observations, is dominated by the strong subsurface cooling located in Northwest Pacific Subtropical Gyre (NPSG) region within 15 °N–35 °N. This subsurface cooling is accompanied by a strong freshening, as indicated in Fig. 8, that is due to the ventilation of surface signals into the subsurface layer, as documented in several recent studies^44^,^45^. The heaving effect^46^,^47^, which is an adiabatic fluid motion associated with the adjustment of wind-driven circulation, could provide one explanation for the large-scale geographical pattern of temperature and salinity changes from 301 to 1500 m (shown in Figs 3 and 8). The stronger easterly winds that have appeared in low latitudes (i.e., 30 °S ∼ 30 °N in the Pacific Ocean) since the late 1990s have pumped cold and salty water from the deeper ocean to the upper ocean^47^, resulting in the cooling and salinification of the Pacific Ocean at low latitudes (i.e., 301–1500 m in Figs 3 and 8). Meanwhile, it inputs the warm/fresh water in high latitudes (i.e., 30 °N ∼ 50 °N, 30 °S ∼ 50 °S) into the deeper ocean (i.e., 301–1500 m in Figs 3 and 8). We note here that these explanations are the authors’ hypotheses, and fully addressing these hypotheses requires more careful studies.

Furthermore, we showed that the CMIP5 models are capable of simulating such low frequency variabilities on 10-years scales (i.e., SST-Hiatus and 301–700 m Slowdown). However, numerous previous examinations of CMIP5 20th-century simulations indicate that most of the models do not predict recent climate hiatus^5^,^11^,^35^, suggesting that the models may have limited ability to reconstruct the timing of the phase changes.

Finally, the existence of the slowdown periods (due to natural variability) does not contradict the fact of global warming. However, the ocean response to radiative forcing is still unclear, and all of the four observation products reflect a net warming of the ocean column (1–1500 m) since 2004, at 0.27 ± 0.06 W/m^2^ (EN4), 0.38 ± 0.07 W/m^2^ (Ishii), 0.40 ± 0.10 W/m^2^ (IGOT) and 0.28 ± 0.07 W/m^2^ (Argo) per unit area of earth’s surface, which is consistent with previous observation-based estimations^18^,^26^,^28^. Approximately 50–70% (different over 4 datasets) of the ocean-column warming is encompassed by the deep ocean warming for 701–1500 m (in Fig. 1a,b). Although the ocean has experienced heat redistribution from 1–100 m to 101–300 m and cooling of 301–700 m, the net ocean temperature change for 1–700 m is a warming, which contributes to the other 30–50% of the ocean-column warming within 1–1500 m. It is still unclear how the heat is being transferred to the deeper ocean (i.e., 700–1500 m). A hypothesis has been proposed in a recent study^15^ highlighting the importance of the Atlantic Ocean in a much longer time scale of 20–30 years. The net ocean warming reflects the existence of climate change in the ocean, which acts as a response to the radiative forcing, helps transport heat from the sea surface to the deeper ocean, and results in net ocean warming. Therefore, we conclude that both internal variability and external forcing have controlled the evolution of the interior ocean temperature in this century and that the recent climate hiatus is not an unusual climate phenomenon.

## Methods

### Observations

The ocean temperature observations are obtained from the World Ocean Database 2009^48^ from 1966 to 2013. Only measurements with “Good” flags, indicating high quality, are used. Argo data are sourced from the FTP server of the Argo Science team (ftp://usgodae1.fnmoc.navy.mil/pub/). For Argo data, only temperature and pressure measurements of the delayed mode with flag = 1 are used. A new correction scheme^49^ with an Expendable BathyThermograph (XBT) bias is applied to correct the historical XBT dataset. The Mechanical BathyThermograph (MBT) bias is corrected by using a scheme proposed by ref. 23. All the temperature measurements are passed through quality-control processes. This new bias-corrected and quality-controlled dataset is named the “Institute of Atmospheric Physics (IAP) Global Ocean Temperature (IGOT) dataset, version 1.0” (hereafter, the IGOT dataset).

For the IGOT dataset, to calculate ocean temperature changes, we first choose a specific monthly climatology constructed by using all observations from 2008 to 2012 (named 2008–2012Clim). This climatology is subtracted from each temperature profile to obtain a temperature anomaly. All the temperature anomaly profiles are first interpolated to standard vertical levels and then grouped into a 5 ° by 5 ° spatial grid and a one-month temporal bin. The grid-averaged temperature anomaly profile is obtained in each grid box as the arithmetic mean.

Three independent objectively analyzed ocean subsurface temperature products are used in this study to evaluate our key results. The first, from ref. 23, used all the ocean subsurface temperature observations, and an XBT bias correction scheme proposed by the authors was applied. The second is an Argo gridded dataset^50^ where only Argo data are used and objectively analyzed. Finally, the third dataset is the EN4 objective analysis dataset^22^, which also used all subsurface temperature observations. The three datasets are all examined under objective analyses to obtain a full global map of the subsurface temperature field.

### Models

Twenty-three models from the Coupled Model Inter-comparison Project Phase 5 (CMIP5) project are collected and used in this study^37^. Information on the models we used is presented in Table S1 in the supporting information. Experiments of the control run with pre-industrial CO_2_ forcing (piControl) are used because the effect of greenhouse gases on the ocean changes must be excluded to identify the natural variability and to generate enough data from an extended time period. At least 200 years of model runs are collected for each model, and most of them contain 500 years of data (Table S1 in the supporting information). To minimize the impacts of the climate shift in CMIP5 models^51^, which appears as spurious long-term changes independent of either inertial variability or responses to external forcing, we simply removed a linear long-term trend before our analyses.

The method for constructing the composite is similar to that presented in ref. 4. In brief, to construct the composite in the SST-Hiatus, the annual global-averaged SST is calculated. We then calculated the 10-year SST trends starting from each model year. Negative values represent global sea surface cooling, and the peaks of the trends are marked. All peaks with negative values smaller than −1 standard deviation of the SST time series are selected, and the corresponding 10-year trend is denoted as a SST-Hiatus period. A single composite is obtained by averaging the temperatures at all the SST-Hiatus periods. The composite for the 301–700 m Slowdown period is obtained similarly by using the 301–700 m averaged temperature time series as an indicator.

### Significance of trend and correlation

A modified Mann-Kendall test is applied for the significance of anomalies of trends, which is a non-parametric and rank-based test widely used in meteorology studies. Because the method is rank based, extreme data points in the time series will not greatly affect the results of the trend analysis. The traditional Mann-Kendall test is modified as described in ref. 52 to remove the impact of autocorrelation. When calculating correlation, the Pearson *r* correlation is used, and the significance is tested by a traditional *t*-test.

## Additional Information

**How to cite this article**: Cheng, L. *et al.* Distinctive ocean interior changes during the recent warming slowdown. *Sci. Rep.* **5**, 14346; doi: 10.1038/srep14346 (2015).
